# Terahertz Properties of GeAsSeSbSnTe Chalcogenide Glassy Semiconductors

**DOI:** 10.3390/mi17050533

**Published:** 2026-04-27

**Authors:** Alexander V. Andrianov, Alexey A. Shakhmin, Alexey G. Petrov, Nikolay V. Sivov, Grigory I. Kropotov

**Affiliations:** 1 Ioffe Institute, St. Petersburg 194021, Russia; alex.andrianov@mail.ioffe.ru (A.V.A.); petrov@mail.ioffe.ru (A.G.P.); 2 Tydex LLC, St. Petersburg 194292, Russia; alexeyshakhmin@tydex.ru (A.A.S.); grigorykropotov@tydex.ru (G.I.K.)

**Keywords:** chalcogenide glasses, amorphous and highly disordered materials, terahertz spectra, refractive index, extinction coefficient

## Abstract

Chalcogenide glasses are known as optical materials for the infrared spectral range. These compounds may also be of interest as materials for the low-frequency part of the terahertz range of electromagnetic waves, which is currently being intensively studied in connection with the numerous possible applications of terahertz radiation. However, the terahertz optical characteristics of chalcogenide glasses remain poorly studied. In this work, eight different compositions of GeAsSeSbSnTe chalcogenide glasses were investigated using terahertz time-domain spectroscopy. A number of compositions, in particular GeSeTe and AsSeSbSn, were studied in the terahertz spectral range for the first time. Spectra of the refractive index and extinction coefficient were obtained for studied materials in the spectral range of 0.1–2.2 THz. The experimental frequency dependence of the product of the terahertz power absorption coefficient and the refractive index for the entire set of studied glasses is approximated by a power function. It was established that the exponent of the approximating power functions varies from 1.68 to 2.34 depending on the composition of the chalcogenide glass. For the studied glasses, a correlation was found between the values of the average coordination number characterizing the chalcogenide glass structure, and the values of the exponent of the functions approximating the THz absorption spectra.

## 1. Introduction

In the last few decades, the direction associated with research in the field of electromagnetic waves of the terahertz (THz) frequency range (with frequencies from 0.1 to 10 THz) has undergone rapid development, which is due to the prospects for the application of THz radiation in various fields of science and technology [1]. At the same time, the intensity of both fundamental research in the field of THz electromagnetic waves and applied research and development of devices for the THz spectrum range is continuously increasing [2]. A significant part of the research is aimed at finding ways to create efficient sources [3,4] and sensitive detectors of THz radiation [5]. An important direction of THz research is the search for materials suitable for the construction of THz optical systems and the development of technology for creating THz optics elements [6,7].

**Figure 1:**
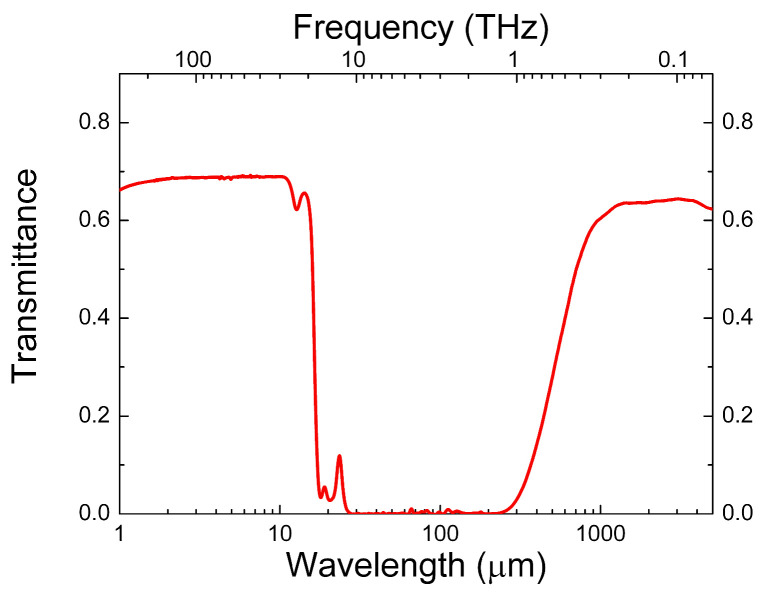
Characteristic transmittance spectrum of a 2 mm glass plate based on the compound. Ge_0.314_As_0.118_Se_0.568_. Measurements were done using Bruker Vertex 70 Fourier transform spectrometer.

From this point of view, chalcogenide glasses are promising materials. Chalcogenide glasses are based on chalcogens, elements from Group 6 of the periodic table of elements, such as S, Se, and Te. When combined with other elements, such as Ge, As, and Sb, they form stable glasses with semiconductor properties [8,9]. In addition, such materials have a transparency window in the IR wavelength range (1–14 μm) and are used to create IR optical elements [10,11]. At the same time, chalcogenide glasses can be transparent at wavelengths greater than 500 μm (or at frequencies below 0.6 THz). Figure 1 demonstrates the transmittance spectrum of GeAsSe glass over a wide spectral region, including the mid-IR and THz ranges.

Therefore, chalcogenide glasses can also be used to create optical elements (various lenses, windows, prisms, splitter plates, substrates, etc.) operating in the low-frequency part of the THz range, as well as in the sub-terahertz frequency range. Despite the great interest in these materials, caused by the prospects of their application for creating THz optical elements, the optical characteristics of such materials in the THz spectral region remain poorly studied. Studies of their THz properties are limited to only a few works, among which articles [12,13,14,15] can be highlighted.

In the present work, we investigate the optical characteristics of chalcogenide glasses Ge_x_As_y_Se_1−x−y−z−s−t_Sb_z_Sn_s_Te_t_ of eight different compositions using the THz time-domain spectroscopy (THz-TDS) method. The materials selected for the studies were compounds, based on two-, three-, or four-element compositions with different stoichiometries. Such materials are of interest not only for applications in IR optics, but also in sub-THz range optics, and have optical properties similar to those of chalcogenide glass, the transmittance spectrum of which is shown in Figure 1. In addition, such materials are available for purchase from various manufacturers, including VITAL, NHG, UMICORE, CDGM and SCHOTT. In the spectral range of 0.1–2.2 THz, spectra of the refractive index, extinction coefficient as well as the THz power absorption coefficient were obtained for the entire set of studied glasses. The THz absorption coefficient has strong frequency dependence and this dependence can be approximated by a power function. A similar pattern is characteristic of a wide range of amorphous and glassy materials [16,17].

## 2. Materials and Methods

Chalcogenide glass (CG) materials for research were purchased from VITAL (Guangzhou, China). Eight different compositions of chalcogenide glasses were investigated, which were systems consisting of two, three or four elements with different stoichiometries. The types and compositions of materials are indicated in Table 1. To conduct THz studies, samples were prepared in the form of optically polished plane-parallel plates with a diameter of 25 mm and a set of thicknesses from 1 to 2 mm. The surface roughness of the prepared samples was no worse than 50 nm, which was ensured by the chemical–mechanical optical processing technology developed by Tydex LLC (St. Petersburg, Russia). The degree of roughness was controlled using laser interferometry on test samples processed within the existing technology.

**Figure 2:**
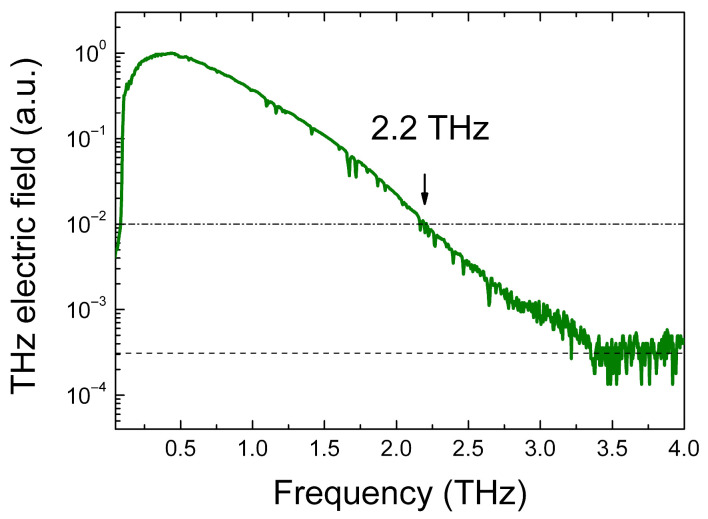
Amplitude spectrum of the reference THz signal (without a sample under test) in the developed coherent THz spectrometer. The THz signal is normalized to a maximum. Spectral resolution is 8 GHz. The dashed line corresponds to the noise floor, and the dashed-dotted line corresponds to a signal level of 0.01 from the maximum.

The transmission of THz radiation at normal incidence onto the chalcogenide glass plates was studied. The measurements were carried out on a THz coherent spectrometer built on the base of a femtosecond C-Fiber laser generating radiation with a wavelength of 780 nm, pulse duration of 90 fs, repetition rate of 100 MHz, and an average laser power of approximately 450 mW. Photoconductive antennas based on LT-GaAs were used as a source and detector of THz radiation. The internal volume of the spectrometer was purged with dry nitrogen. The working spectral range of the created coherent THz spectrometer at a level of 0.01 from the THz signal maximum is 0.1–2.2 THz (see Figure 2).

The complex amplitude THz transmission coefficient of a plane-parallel sample has the following form [18]:

(1) $$\tilde{T}(\omega )=T(\omega )e^{i\phi (\omega )}=\frac{E_{s}(\omega )}{E_{ref}(\omega )}=\frac{4\tilde{n}}{{\left(\tilde{n}+1\right)}^{2}}\times \frac{\exp[\frac{i\omega }{c}d(\tilde{n}-1)]}{\left[1-\frac{{\left(\tilde{n}-1\right)}^{2}}{{\left(\tilde{n}+1\right)}^{2}}\exp(2\frac{i\omega }{c}d\tilde{n})\right]}$$

where $T(\omega )=\left|\frac{E_{s}(\omega )}{E_{ref}(\omega )}\right|$ is the amplitude transmission spectrum, ϕ(ω) is the phase transmission spectrum, $E_{ref}(\omega )$ and $E_{s}(\omega )$ are, respectively, the complex amplitudes of the signals of the reference THz radiation and the THz radiation transmitted through the sample, which can be obtained by Fourier transforming the corresponding wave forms, $\tilde{n}=n+ik$ is the complex refractive index of the material with n and k being, respectively, the refractive index and the extinction coefficient, d is the thickness of the sample, ω=2πf, where f is the frequency of the THz radiation, and c is the speed of light. The denominator in square brackets on the right-hand side of Equation (1) describes the interference of THz radiation in the sample. In the case of strong absorption of THz radiation, typical for the materials under study, interference can be neglected and the denominator assumed to be equal to one. Furthermore, in the spectral region of the THz spectrometer used, the inequality n>>k (see Section 3) exists for the materials under study. Taking this into account, Equation (1) can be rewritten as:

(2) $$\tilde{T}(\omega )=T(\omega )e^{i\phi (\omega )}=\frac{4n}{{\left(n+1\right)}^{2}}\times \exp[\frac{i\omega }{c}d(n-1)]\times \exp(-\frac{\omega }{c}kd)$$

From Equation (2) it directly follows that

(3) $$\phi (\omega )=\frac{\omega }{c}d[n(\omega )-1]$$

(4) $$T(\omega )=\frac{4n}{{\left(n+1\right)}^{2}}\times \exp[-\frac{\omega }{c}k(\omega )d]$$

Thus, having an experimental phase spectrum of the THz transmission and using Equation (3), the refractive index spectrum of the material under study can be obtained. Further, using Equation (4) and having an experimental amplitude transmission spectrum, the extinction coefficient spectrum k(ω) can be obtained, which is related to the THz power absorption coefficient, α, by the formula $\alpha =\frac{2\omega }{c}k$. Using this method, the THz spectra of the quantities n and k, as well as α, were determined for the chalcogenide glasses investigated.

**Figure 3:**
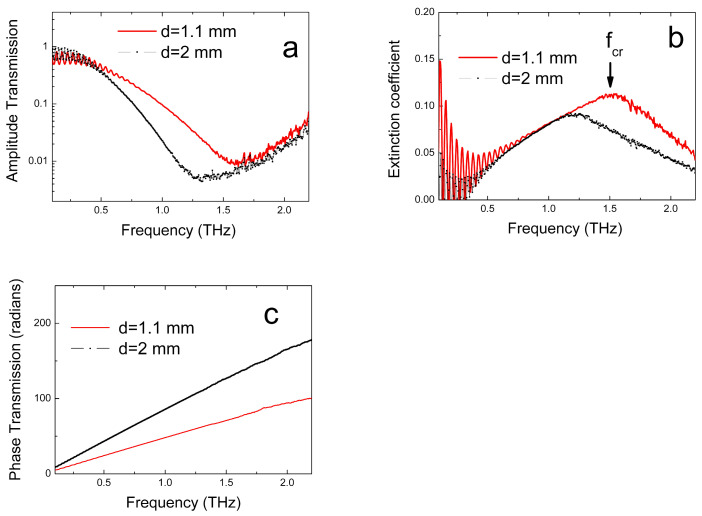
(**a**) THz amplitude transmission spectra of VIG06 (As_0.387_Se_0.613_) glass samples with thicknesses of 2- and 1.1-mm. (**b**) THz extinction coefficient spectra of VIG06 glass samples based on the THz transmission measurement data and their processing using Equations (2)–(4). The arrow indicates the frequency $f_{cr}$, up to which the values of the extinction coefficient in the spectrum of a sample with a thickness of 1.1 mm (the thinnest of the two measured) can be considered correct. (**c**) THz phase transmission spectra of VIG06 glass samples with thicknesses of 2- and 1.1-mm.

A distinctive feature of the glasses studied in this work is that these materials have very strong absorption at frequencies above 1 THz. Figure 3a shows the THz amplitude transmission spectra for VIG06 (A_s0.387_Se_0.613_) glass samples with thicknesses of 2 and 1.1 mm.

The spectrum shows a minimum in the transmission coefficient, which, however, is not due to a resonance in the absorption spectrum of the material, since its position depends on the sample thickness, but is an artifact caused by a combination of strong THz absorption in the studied material and the limited bandwidth of the THz spectrometer used (0.1–2.2 THz). The cause of such artifacts in THz-TDS transmission studies in strongly absorbing materials was quantitatively analyzed in [19]. Figure 3b shows the extinction coefficient spectra for VIG06 glass samples, obtained using the method described. Oscillations in the spectra at frequencies below 0.7 THz are due to the interference of radiation in the samples, which is not considered in Equations (2)–(4). The maximum in the spectrum of k magnitude is also associated with the manifestation of an artifact caused by strong absorption in the studied material and the limited spectral range of the THz spectrometer. Nevertheless, at frequencies below $f_{cr}$, (the frequency near the maximum in the spectrum of k), the values of the extinction coefficient can be considered correct [19]. It should be noted that $f_{cr}$ will increase with decreasing the sample thickness and, thus, by decreasing the thickness of the plates under study, it is possible to reach higher frequencies in the THz spectrum of the k magnitude of the material.

It should be noted also that the determination of the optical characteristic n using Equation (3) does not include the amplitude transmission T(ω) and, therefore, the associated artifact discussed above does not appear at frequencies exceeding $f_{cr}$. From Figure 3c one can see that THz phase transmission spectra, ϕ(ω), are smooth and have no specific features in the frequency range of $f_{cr}$, or higher, and the THz phase transmission spectrum itself increases proportionally to d. Therefore, the THz transmission phase values, as well as the refractive index spectrum determined from them, can be considered correct over the entire frequency range of the THz spectrometer used (0.1–2.2 THz).

## 3. Experimental Results and Discussion

**Figure 4:**
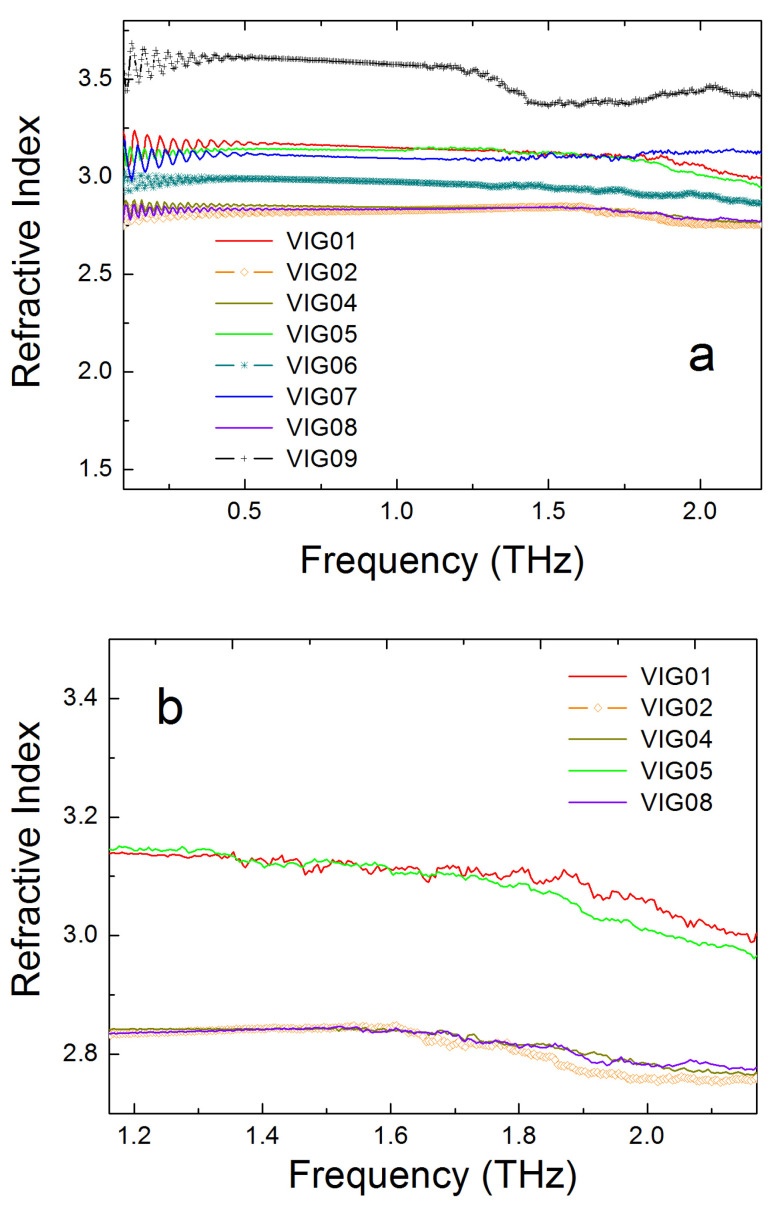
(**a**) Refractive index spectra for a series of studied GeAsSeSbSnTe chalcogenide glass samples with a thickness of approximately 1 mm. (**b**) The magnified portion of the refractive index spectra for several glasses. Compositions of CG glasses: VIG01—As_0.279_Se_0.617_Sb_0.06_Sn_0.044_, VIG02—Ge_0.314_As_0.118_Se_0.568_, VIG04—Ge_0.095_As_0.39_Se_0.515_, VIG05—Ge_0.247_Sb_0.178_Se_0.575_, VIG06—As_0.387_Se_0.613_, VIG07—Ge_0.173_Sb_0.217_Se_0.61_, VIG08—Ge_0.208_As_0.195_Se_0.597_, VIG09—Ge_0.16_Se_0.1_Te_0.74_.

Figure 4 shows the refractive index spectra for a set of the studied chalcogenide glasses. As can be seen from the figure, the refractive index in the studied glasses has a very weak dependence on frequency. At a frequency of about 1 THz, its values range from 2.8 to 3.6, depending on the material composition. The refractive index values are close to those characteristics of semiconductor crystals such as Si or GaAs [20]. The obtained data can be used in the design of optical elements for the sub-THz and THz ranges and, particularly, in the design of various lenses. Oscillations in the n spectra, noticeable at frequencies lower than ~0.5 THz, are due to the interference effect in plane-parallel samples, which is not considered in Equations (2) and (3) used in processing our experimental data. It can also be seen that for some compositions of the studied chalcogenide glasses there is a slight decrease in the refractive index with increasing frequency (see also Figure 4b), i.e., anomalous dispersion is present. The fact of the existence of anomalous dispersion in chalcogenide glasses in the THz spectral region was noted in [13].

**Figure 5:**
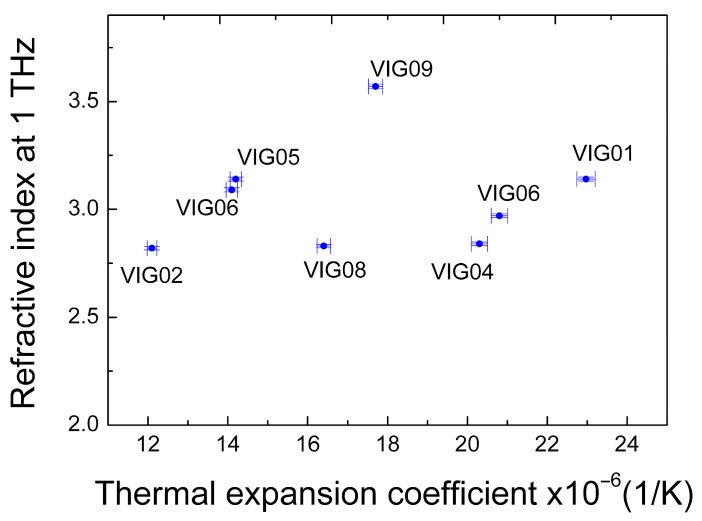
The variation in the refractive index at 1 THz with the thermal expansion coefficient for the set of studied CG glasses. Thermal expansion coefficients are given for the temperature range of 293–393 K. Chalcogenide glass compositions: VIG01—As_0.279_Se_0.617_Sb_0.06_Sn_0.044_, VIG02—Ge_0.314_As_0.118_Se_0.568_, VIG04—Ge_0.095_As_0.39_Se_0.515_, VIG05—Ge_0.247_Sb_0.178_Se_0.575_, VIG06—As_0.387_Se_0.613_, VIG07—Ge_0.173_Sb_0.217_Se_0.61_, VIG08—Ge_0.208_As_0.195_Se_0.597_, VIG09—Ge_0.16_Se_0.1_Te_0.74_.

In [21], during a study of the THz properties of several amorphous and glassy materials, a strong correlation was established between the THz refractive index and the thermal expansion coefficient of the materials. For the CG glasses studied in the present work, we also compared the measured values of the THz refractive index with the data on the thermal expansion coefficients provided by the VITAL. However, no correlation was observed between these values for the set of glasses studied (see Figure 5).

**Figure 6:**
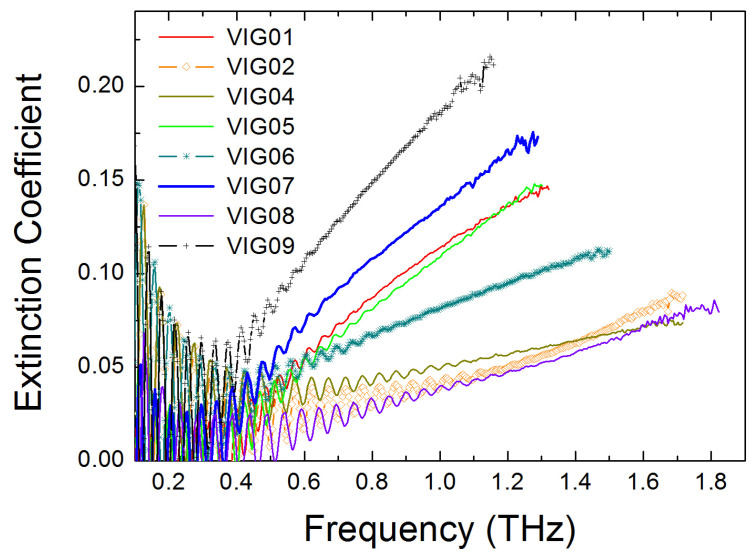
THz extinction coefficient spectra for a set of studied chalcogenide glasses. Compositions of glasses: VIG01—As_0.279_Se_0.617_Sb_0.06_Sn_0.044_, VIG02—Ge_0.314_As_0.118_Se_0.568_, VIG04—Ge_0.095_As_0.39_Se_0.515_, VIG05—Ge_0.247_Sb_0.178_Se_0.575_, VIG06—As_0.387_Se_0.613_, CG07—Ge_0.173_Sb_0.217_Se_0.61_, VIG08—Ge_0.208_As_0.195_Se_0.597_, VIG09—Ge_0.16_Se_0.1_Te_0.74_.

Figure 6 shows the extinction coefficient spectra of the studied chalcogenide glasses. The data were obtained on samples with a thickness of about 1 mm and are limited to frequencies below $f_{cr}$, which were determined for each of the studied samples.

Oscillations in the spectra observed at low frequencies are also caused by the THz radiation interference effect. The extinction coefficients and, accordingly, the THz power absorption coefficients, α, in the studied materials increase strongly with frequency, which is consistent with the results of [12,13,14,15]. In addition, the extinction coefficient values are higher in glasses with higher refractive index values. Following the approach used, for example, in [12,14,15], it is possible to approximate the quantity nα by a power function of frequency, that is, a dependence of the form $n\alpha =Kf^{b}$, where K and b are constants.

**Figure 7:**
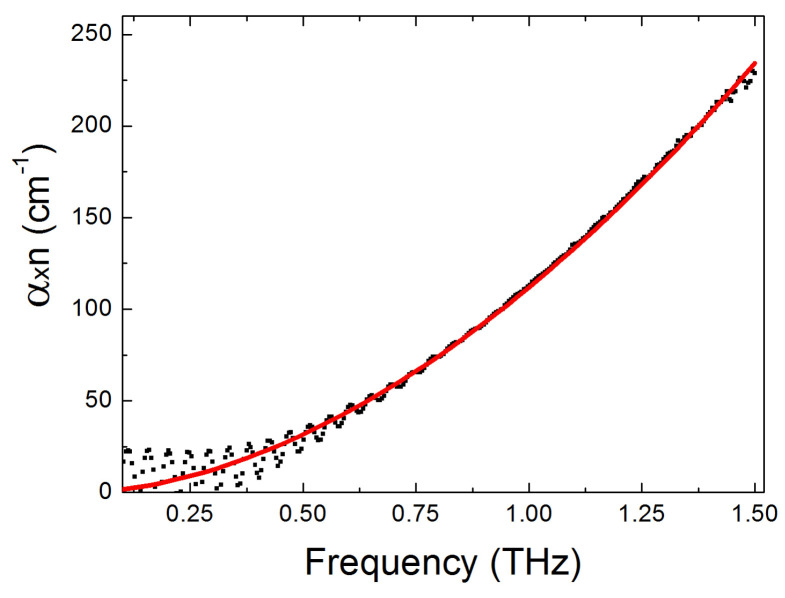
An example of approximation of the THz spectrum of nα (experimental points) by a power function of frequency of the form $n\alpha =Kf^{b}$, (solid curve), where f is the frequency in THz, K and b are constants, for a sample of chalcogenide glass VIG06 (As_0.387_Se_0.613_) with a thickness of 1.1 mm. The parameters of the best curve-fitting are as follows: K = 111.85 ± 0.41 cm^−1^, b = 1.82 ± 0.01.

Figure 7 shows an example of such an approximation for chalcogenide glass VIG06. The approximation was carried out using the least squares method.

It can be seen that the power law $n\alpha =Kf^{b}$ describes the experimental data quite well. A power law of this type for the parameter nα has been observed for a wide class of highly disordered systems, such as amorphous and glassy materials (see, for example, [16,17]) and is apparently due to the disorder-induced absorption of THz electromagnetic radiation at low-frequency vibrational modes associated with charged defects or with disorder-induced charge fluctuations [16,17,22]. This approximation of the experimental data was performed using the least squares method for the entire set of chalcogenide glasses studied, and the results are presented in Table 2.

For the materials studied in this work, these parameters K and b are generally in the order of magnitude consistent with the results obtained for other amorphous and glassy materials [14,15]. At the same time, the parameters b for some material compositions, particularly for VIG02 (Ge_0.314_As_0.118_Se_0.568_) and VIG05 (Ge_0.247_Sb_0.178_Se_0.575_), significantly exceed the value b = 2, which was established for chalcogenide glasses in several studies (see, e.g., [12,16,17]).

**Figure 8:**
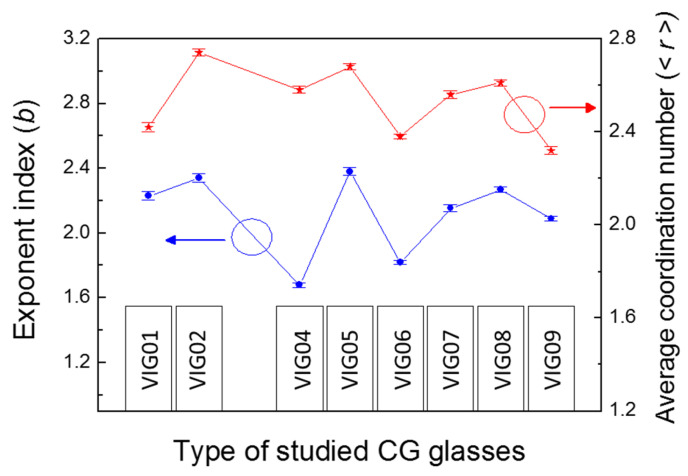
Calculated values of the average coordination number <r> (red stars) and experimental values of the parameter b (blue circles) characterizing the THz absorption for the set of studied chalcogenide glasses GeAsSeSbSnTe. Blue arrow pointing to the left vertical axis indicates the values of the b parameter. The red arrow pointing to the right vertical axis indicates the values of the <r> parameter. Compositions of CG glasses: VIG01—As_0.279_Se_0.617_Sb_0.06_Sn_0.044_, VIG02—Ge_0.314_As_0.118_Se_0.568_, VIG04—Ge_0.095_As_0.39_Se_0.515_, VIG05—Ge_0.247_Sb_0.178_Se_0.575_, VIG06—As_0.387_Se_0.613_, CG07—Ge_0.173_Sb_0.217_Se_0.61_, VIG08—Ge_0.208_As_0.195_Se_0.597_, VIG09—Ge_0.16_Se_0.1_Te_0.74_.

In [14], a hypothesis was put forward that glasses with values of the parameter b significantly higher than 2 belong to materials with higher values of average coordination numbers <r>, corresponding to the average number of chemical bonds per atom in the material. For covalent glasses, which include chalcogenide glasses, the value <r> can be calculated using the “8-N” rule [23,24,25]:

(5) $$<r>=8-\sum_{i}x_{i}N_{v}^{x_{i}}$$

where $x_{i}$ is the atomic fraction of the *i*-th component in the glass and $N_{v}^{x_{i}}$ is the number of valence electrons of the *i*-th element. The average coordination numbers <r> were calculated for the set of chalcogenide glasses studied in this work. Figure 8 shows the results of these calculations. The values of the parameter b determined from the THz absorption experiment are also presented here.

Figure clearly shows that a correlation does indeed exist between the average coordination numbers <r> in the materials, reflecting the structure of the chalcogenide glass, and the values of the parameter b, characterizing the THz absorption spectrum in the chalcogenide glasses studied. These values change almost synchronously.

The observed correlation between parameters <r> and b can be qualitatively explained as follows. For values of the average coordination number <r> in the range between the values corresponding to a perfect crystal lattice (for example, values of <r> of the order of 4 correspond to perfect covalent crystals), the <r> parameter reflects the degree of disorder of the material. Accordingly, under these conditions, with an increase in the <r> magnitude, the system becomes more disordered. This in turn leads to a higher density of low-frequency vibrational modes and a stronger dependence of their density on energy. It is on such vibrational modes that the absorption of THz radiation occurs.

## 4. Conclusions

For a set of Ge_x_As_y_Se_1−x−y−z−s−t_Sb_z_Sn_s_Te_t_ chalcogenide glasses of eight different compositions, spectra of extinction coefficient and refractive index were obtained in the 0.1–2.2 THz frequency range. The refractive index of the studied glasses exhibits a weak spectral dependence, and its values at a frequency of about 1 THz vary from 2.8 to 3.6, depending on the material composition. The observed THz refractive index values are close to those of crystals of a wide range of semiconductors. The extinction coefficient increases significantly with increasing frequency. Moreover, the extinction coefficient values are higher in glasses with higher refractive indices. The spectra of the product of the THz power absorption coefficient α and the refractive index n can be approximated by power functions. Such patterns have been observed for a wide class of highly disordered systems, such as amorphous and glassy materials. The exponent b for the approximating power functions varies from 1.68 to 2.38 depending on the composition of the chalcogenide glass. A correlation was found between the values of the average coordination number <r>, which characterizes the chalcogenide glass structure, and the parameter b, which characterizes the THz absorption, for the studied GeAsSeSbSnTe chalcogenide glasses. In addition, in the present work, a number of chalcogenide glass compositions, namely GeSeTe and AsSeSbSn were investigated in the THz spectral range for the first time.

## Figures and Tables

**Table 1:** Types of studied glasses and their compositions.

Type of CG	Composition
VIG01	As_0.279_Se_0.617_Sb_0.06_Sn_0.044_
VIG02	Ge_0.314_As_0.118_Se_0.568_
VIG04	Ge_0.095_As_0.39_Se_0.515_
VIG05	Ge_0.247_Sb_0.178_Se_0.575_
VIG06	As_0.387_Se_0.613_
VIG07	Ge_0.173_Sb_0.217_Se_0.61_
VIG08	Ge_0.208_As_0.195_Se_0.597_
VIG09	Ge_0.16_Se_0.1_Te_0.74_

**Table 2:** The values of frequencies $f_{cr}$, as well as the values of parameters K and b, obtained by approximation the experimental THz spectra of nα with a power function $n\alpha =Kf^{b}$, for the set of studied chalcogenide glass GeAsSeSbSnTe samples with a thickness of about 1 mm.

Type of CG	Composition	$f_{cr}$ (THz)	K (cm^−1^)	b
VIG01	As_0.279_Se_0.617_Sb_0.06_Sn_0.044_	1.32	143.54 ± 0.81	2.23 ± 0.03
VIG02	Ge_0.314_As_0.118_Se_0.568_	1.71	49.84 ± 0.54	2.34 ± 0.03
VIG04	Ge_0.095_As_0.39_Se_0.515_	1.71	61.34 ± 0.35	1.68 ± 0.01
VIG05	Ge_0.247_Sb_0.178_Se_0.575_	1.30	140.74 ± 0.67	2.38 ± 0.02
VIG06	As_0.387_Se_0.613_	1.50	111.85 ± 0.41	1.82 ± 0.01
VIG07	Ge_0.173_Sb_0.217_Se_0.61_	1.29	172.48 ± 0.68	2.15 ± 0.02
VIG08	Ge_0.208_As_0.195_Se_0.597_	1.82	45.11 ± 0.39	2.27 ± 0.02
VIG09	Ge_0.16_Se_0.1_Te_0.74_	1.16	277.51 ± 0.89	2.09 ± 0.02

## Data Availability

The original contributions presented in this study are included in the article. Further inquiries can be directed to the corresponding author.
